# Integrated Network Analysis Decipher ZNF384‐Related miR‐20b‐5p and miR‐424‐5p in Colon Adenocarcinoma

**DOI:** 10.1002/cnr2.70233

**Published:** 2025-05-22

**Authors:** Bo Zhang, Yoshihisa Matsumoto

**Affiliations:** ^1^ Department of Transdisciplinary Science and Engineering School of Environment and Society, Institute of Science Tokyo Tokyo Japan; ^2^ Laboratory for Zero‐Carbon Energy, Institute of Integrated Research Institute of Science Tokyo Tokyo Japan

**Keywords:** bioinformatics, colon adenocarcinoma, miR‐20b‐5p, miR‐424‐5p, weighted gene co‐expression network analysis (WGCNA), ZNF384

## Abstract

**Background:**

ZNF384 is a C2H2‐type zinc finger protein (ZNF) which is implicated in DNA double‐strand break (DSB) repair through the classical non‐homologous end‐joining (cNHEJ) pathway.

**Aims:**

To clarify the regulatory mechanisms involving ZNF384 in colon adenocarcinoma (COAD).

**Methods and Results:**

First, we conducted a differential expression gene (DEG) analysis of mRNA and lncRNA using TCGA‐COAD RNA‐Seq data. We also identified ZNF384‐related mRNAs through Pearson's correlation coefficient calculation and conducted weighted gene co‐expression network analysis (WGCNA) for these genes, leading to the identification of a cluster of 331 genes with strongly positive correlation to tumor, 84 of which overlapped with DEGs. Gene functional analysis showed enrichment of genes in DNA repair, replication fork, and cell cycle checkpoint signaling pathways. Protein–protein interaction (PPI) network analysis of these 84 genes led to the identification of the top 20 key mRNAs. Then we employed three machine learning methods to refine our selection of candidate genes from these intersecting mRNAs. We constructed a competitive endogenous RNA (ceRNA) network and identified two significant intersecting miRNAs, miR‐20b‐5p and miR‐424‐5p, which have been shown to act as a tumor suppressor gene and an oncogene, respectively. Additionally, we found that KIF14 and KIF18B are regulated by these two miRNAs in this ceRNA network, particularly in DNA damage repair and cell cycle. Finally, validation using an external dataset from the GEO database confirmed their expression patterns.

**Conclusion:**

The current study clarifies the mechanisms of how miR‐20b‐5p and miR‐424‐5p work in colon cancer and underscores their predictive capabilities in colon cancer.

## 1 Introduction

Colon cancer shows high morbidity and mortality worldwide, and abnormalities in DNA damage repair mechanisms show significant driving factors in its development [1]. In colon adenocarcinoma (COAD, Table 1), damage to or dysfunction of DNA repair pathways is closely linked to cancer cell proliferation, survival, and resistance to treatment [2].

**TABLE 1:** List of abbreviations.

Abbreviation	Word or phrase
ceRNA	Competing endogenous RNA
cNHEJ	Classical non‐homologous end‐joining
COAD	Colon adenocarcinoma
DDR	DNA damage response
DEGs	Differentially expressed genes
DSB	DNA double‐strand break
FPKM	Fragments per kilobase of transcript per million mapped reads
GO	Gene ontology
KEGG	Kyoto encyclopedia of genes and genomes
LASSO	Least absolute shrinkage and selection operator
lncRNA	Long non‐coding RNA
miRNA	MicroRNA
PPI	Protein–protein interaction
STRING	Search tool for the retrieval of interacting genes/proteins
SVM	Support vector machine
TCGA	The cancer genome atlas
TRRUST	Transcriptional regulatory relationships unraveled by sentence‐based text mining
UCSC	University of California, Santa Cruz
WGCNA	Weighted gene co‐expression network analysis
ZNF	Zinc finger

ZNF384 is a C2H2‐type zinc finger protein (ZNF) involved in the classical non‐homologous end‐joining (cNHEJ) pathway, which is one of the two major DNA double‐strand break (DSB) repair pathways along with homologous recombination (HR). A recent study showed that ZNF384 interacts with the Ku70/Ku80 complex, facilitating its assembly at DNA damage sites and promoting the loading of downstream cNHEJ factors such as APLF and XRCC4/LIG4, thereby enhancing DSB repair efficiency [3].

ZNF384, frequently as fusion proteins with other proteins in leukemia, is shown to be central in the regulatory network of genes related to DNA damage repair, cell cycle, and signaling [4, 5, 6]. ZNF384 is shown to upregulate the expression of Cyclin D1 to promote the proliferation of cancer cells [7]. Cyclin D1 is also shown to be a significant player in DNA damage response (DDR) and repair mechanisms, directly interacting with RAD51, which is a crucial enzyme for HR [8, 9]. Furthermore, recent studies have demonstrated that ZNF384 might promote tumor progression through pathways beyond DDR and cell cycle, such as extracellular matrix remodeling and cancer metastasis [10, 11, 12]. Although the precise mechanisms remain to be clarified, these results suggest that targeting ZNF384 or its downstream effectors could serve as a novel therapeutic approach for cancer.

Nonetheless, the role and mechanism of ZNF384 in COAD have not been fully elucidated, particularly regarding its impact on cancer progression through gene regulation. Although ZNF384 is recognized for its involvement in regulating cellular stress responses and DNA damage repair, the specifics of its role in COAD development and its influence on the cell cycle and DNA damage repair pathways through the regulation of downstream mRNA and miRNA require more in‐depth research. Especially, with which mRNA(s) and/or miRNA(s) ZNF384 interacts and how these interactions function via DNA damage and related protein within the context of colon cancer remain unclear.

These research gaps limit our understanding of the molecular mechanisms of colon cancer, particularly in finding new therapeutic targets and improving cancer treatment strategies. Therefore, by concentrating on the role of ZNF384 in COAD, especially on how it is finely regulated through mRNA, miRNA, and ceRNA networks [13, 14], our study aims to uncover the complex mechanisms of action of this transcription factor in cancer development and to offer new insights and potential targets for the development of targeted therapies.

## 2 Materials and Methods

### 2.1 Differential RNA Expression Analysis in COAD

Five hundred and twelve TCGA‐COAD samples, 41 with adjacent normal tissue samples, were obtained from UCSC Xena [15]. Normal tissue samples were collected from sites at least 2 cm from the tumor margin and were histologically verified to be tumor‐free by pathologists. After removing duplicate samples, our dataset comprised 41 normal samples and 458 tumor samples. After acquiring original count and FKPM files for mRNA, lncRNA, and miRNA, we reannotated these data using the Homo_sapiens.GRCh38.105.chr.gtf.gz file to ensure accurate and updated gene annotations consistent with the GRCh38 reference genome. Because the pre‐normalized UCSC data had been log2‐transformed by adding 1, we reverted it to its original read count format [16]. Genes with zero expression values in over half of the samples were filtered out to refine the gene expression analysis. Subsequently, differential expression analysis of mRNA and lncRNA was conducted using the R package “edgeR” (https://bioconductor.org/packages/release/bioc/html/edgeR.html) and R package “Limma” (https://bioconductor.org/packages/release/bioc/html/limma.html), effectively mitigating sample bias and ensuring robust differential expression analysis [17, 18]. Genes were considered differentially expressed if they exhibited |log(foldchange)| > 1 and *p*‐value < 0.01, indicating significant differences in expression levels.

### 2.2 ZNF384 and Other mRNAs Pearson's Correlation Coefficient Calculation

We performed Pearson's correlation coefficient calculations between the ZNF384 and other mRNAs. We set a threshold of a Pearson's correlation coefficient greater than 0.3 and a *p*‐value < 0.01 to obtain genes that are significantly positively correlated with ZNF384.

### 2.3 WGCNA Construction

WGCNA reveals complex patterns and relationships in large‐scale gene expression data [19]. We established a network based on co‐expression patterns by calculating Pearson's coefficients between gene pairs. After that, we used the dynamic tree division method to establish the basis and cause of the network's expression [20]. Finally, we identified gene modules that are highly associated with tumors by analyzing correlations between modules and external clinical features. We focused on ZNF384 and its co‐expressed mRNAs and explored their roles and potential biological significance in specific cancers. Using the R package WGCNA (v1.72) (https://cran.r‐project.org/web/packages/WGCNA/index.html), we computed adjacency matrices based on Pearson's correlation coefficients between gene pairs to generate a weighted co‐expression network. To achieve a scale‐free topology, we selected a soft thresholding power (*β* = 10) through scale‐free fit analysis (*R* ^2^ > 0.8). Hierarchical clustering with dynamic tree cutting (deepSplit = 2, minimum module size = 30 genes) was applied to identify gene modules, and those with an eigengene correlation greater than 0.75 (dissimilarity threshold = 0.25) were merged to reduce redundancy.

Next, we assessed module‐trait associations by correlating module eigengenes with tumor/normal status using biweight midcorrelation. The red module (*r* = 0.51, *p* = 1 × 10^−23^) exhibited the strongest association with tumor samples and was prioritized for further analysis. Within this module, we focused on ZNF384 and its co‐expressed mRNAs, exploring their potential roles in oncogenic pathways.

### 2.4 Gene Function Analysis and Gene Overlap Analysis

The tumor‐related factors were investigated through WGCNA analysis, along with the analysis of aggregated cancer‐related factors. Overlapping genes were identified and quantified. The significance of overlapping genes was determined using a hypergeometric distribution test with a threshold *p*‐value of less than 0.05. We obtained the genes with a significant correlation between ZNF384 and cancer‐related factors.

We performed functional enrichment analysis for the overlapping gene set using the R package “clusterProfiler” (https://bioconductor.org/packages/release/bioc/html/clusterProfiler.html) to analyze GO, and KEGG pathways [21]. Additionally, we utilized TRRUST (Transcriptional Regulatory Relationships Unraveled by Sentence‐based Text mining) transcription factor prediction tools to identify ZNF384's target genes and further analyze their functions and regulatory relationships [22].

### 2.5 Protein–Protein Interaction (PPI) Interworks to Identify the Hub Genes That Are Related to DNA Damage

We integrated the intersecting mRNA from DEGs and red module genes that were then uploaded to the STRING database (https://string‐db.org) for retrieval of corresponding PPI information [23, 24]. Subsequently, we utilized Cytoscape software (version 3.9.0) to visualize a PPI network among these genes [25, 26]. We employed the Cytoscape plugin “cytoHubba” to enhance the granularity of our network analysis [27].

### 2.6 Machine Learning for Screening the Candidate Genes

We initially selected potential central genes by identifying the intersection of DEGs and key module genes. Subsequently, we employed three machine learning techniques: Least Absolute Shrinkage and Selection Operator (LASSO), Support Vector Machine (SVM), and Random Forest to identify crucial hub genes. As an effective dimensionality reduction method, LASSO demonstrates superior performance over traditional regression analysis when handling high‐dimensional data [28, 29]. We used the R package “glmnet” (https://cran.r‐project.org/web/packages/glmnet/index.html) along with fivefold cross‐validation to perform LASSO analysis with regularization/penalty parameters [30, 31]. In addition, we utilized the “Random Forest” package for random forest analysis, which optimizes the set of variables by assessing the average error rate of candidate central genes, establishes the model, and calculates the importance scores of each gene to determine the top 20 genes of importance [32]. Finally, integrating the results from LASSO, SVM, and Random Forest analyses, we selected characteristic genes specific to tumors.

### 2.7 ceRNA Network Construction and Validation

Interaction pairs of lncRNA–miRNA and miRNA–miRNA were retrieved from the starBase and miRBase databases, respectively [33, 34, 35]. To identify the core genes for network construction, we overlapped genes from the red module, known for their significant correlation with the condition under study, with the list of DEGs. This overlap yielded 84 genes that were present in both datasets. We utilized these genes to construct a ternary interaction network of mRNA–miRNA–lncRNA ceRNA regulatory network visualized and analyzed using Cytoscape. To further verify our research results, we used the GSE39582 dataset in the GEO database for independent verification. Comparing gene expression patterns between disease and control groups revealed regulatory networks consistent with our predictive model.

## 3 Results

### 3.1 Differential Expression Screening of mRNAs and lncRNAs in COAD

**FIGURE 1:**
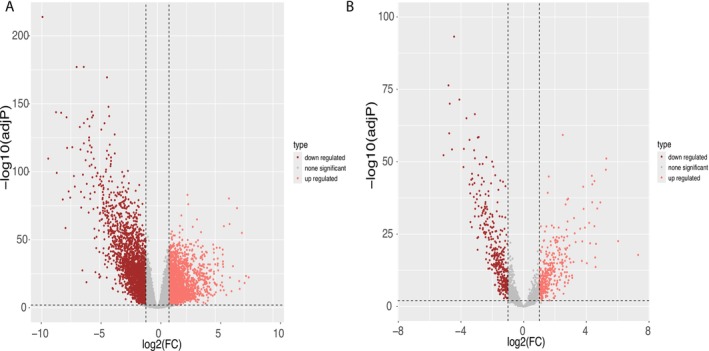
Volcano plot of differentially expressed genes. (A) The volcano plots for differential mRNA. (B) The volcano plots for differential lncRNA.

We initially downloaded COAD RNA expression profiles from the TCGA database to find the genes which are up‐regulated or down‐regulated in tumors as compared to normal tissue. After processing the data as described in Section 2, we obtained 2384 up‐regulated mRNAs and 2474 down‐regulated mRNAs (Figure 1A). We also obtained 363 up‐regulated lncRNAs and 376 down‐regulated lncRNAs in tumors (Figure 1B).

### 3.2 ZNF384 and Other mRNAs Pearson's Correlation Coefficient Calculation

**FIGURE 2:**
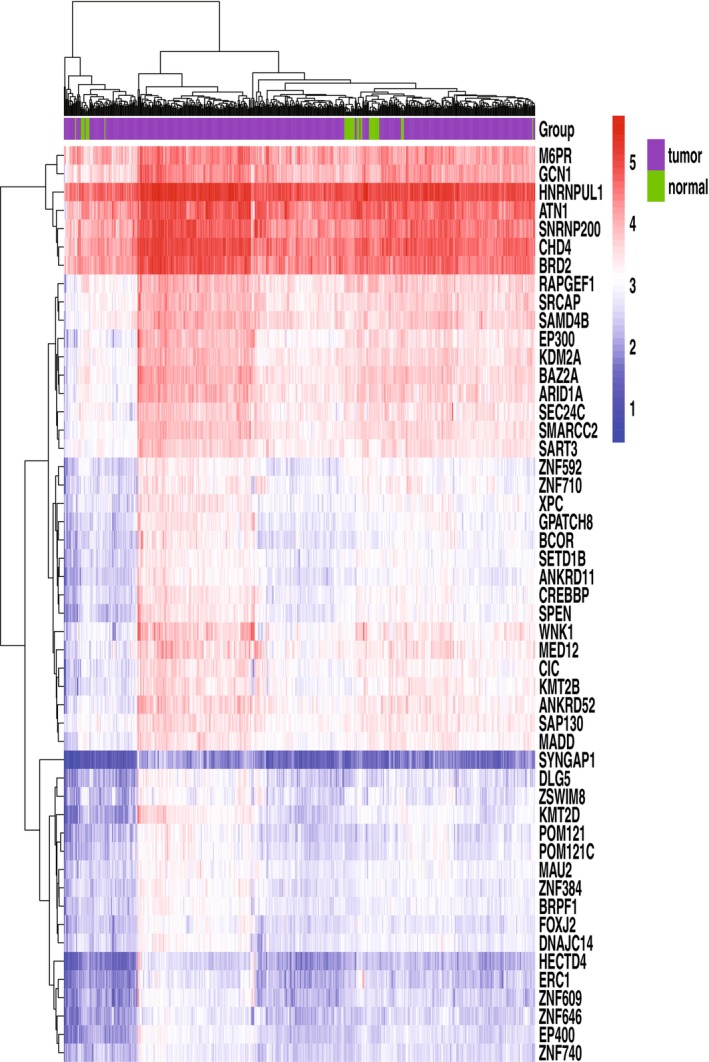
The heatmap of the top 50 ZNF384‐related mRNAs. The heatmap displays hierarchical clustering of the top 50 mRNAs significantly correlated with ZNF384 expression across tumor (labeled 1–5) and normal tissue groups. Rows represent individual mRNAs, and columns correspond to biological samples or replicates. Color intensity reflects normalized expression levels (e.g., red for upregulation and blue for downregulation). Key candidates (e.g., KMT2D, ARID1A, CREBBP, EP300) are highlighted, implicating pathways such as chromatin remodeling, transcriptional regulation, or signaling cascades.

We next sought to find the genes which are regulated by ZNF384. To ensure the relevance and significance of the results, stringent criteria were applied, considering only those mRNAs for which a Pearson's correlation coefficient greater than 0.3 and a *p*‐value less than 0.01 were observed. Through this rigorous filtering process, we identified a set of 3032 mRNAs that displayed a positive correlation with ZNF384. The top ZNF384‐correlated mRNA heatmap (Figure 2) showed the expression level of mRNA in tumor and normal samples. This collection of highly correlated mRNAs constitutes a valuable resource for further investigation into the functional associations and potential regulatory roles of ZNF384 within the context of COAD.

### 3.3 Weighted Gene Co‐Expression Network Analysis (WGCNA)

**FIGURE 3:**
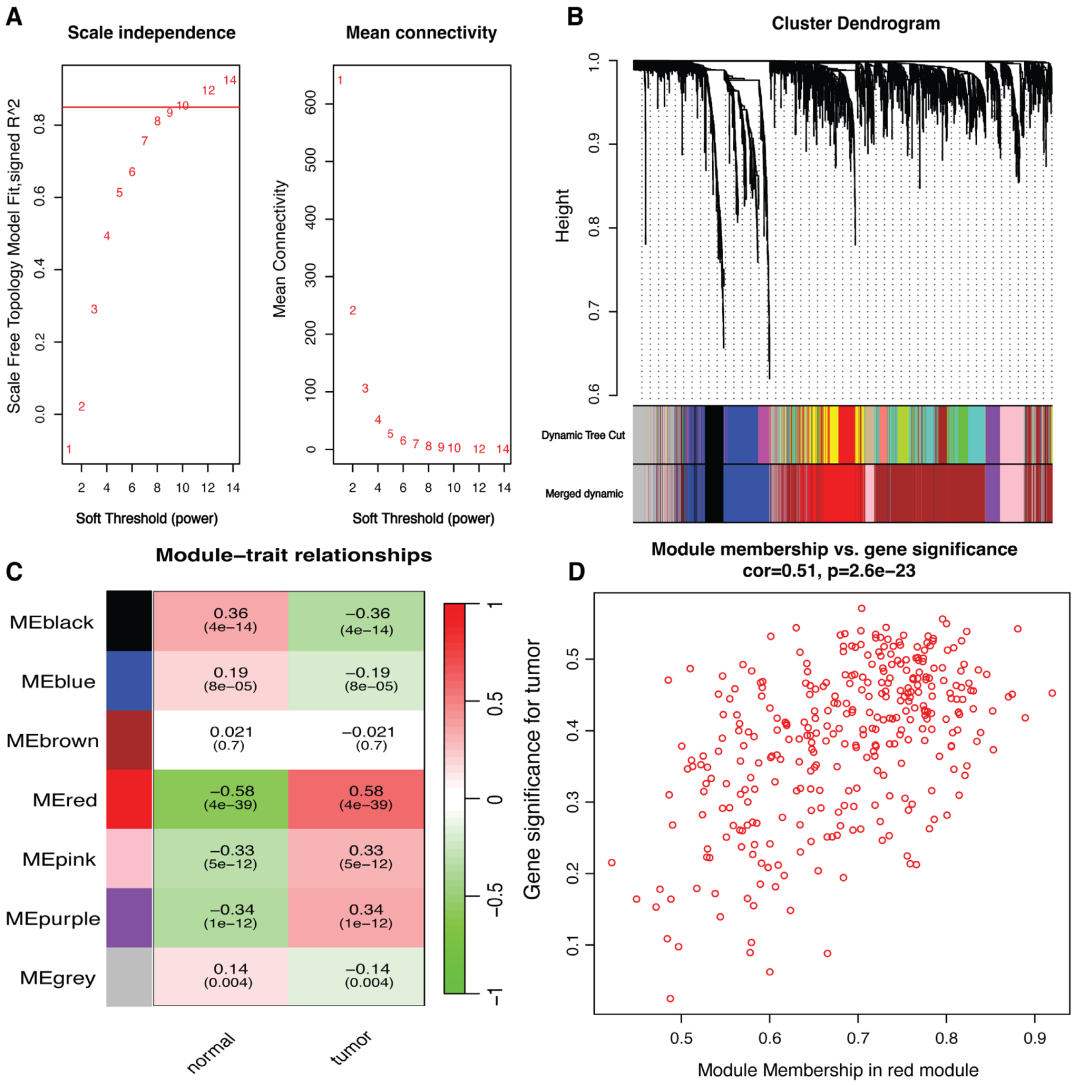
Weighted gene co‐expression network analysis (WGCNA) for identifying key gene modules associated with tumor status. (A) Determination of scale‐free topology criterion. Analysis of scale‐free topology model fit (left) and mean connectivity (right) across different soft‐thresholding powers in WGCNA. The selected soft‐threshold power optimizes network connectivity while approximating a scale‐free topology. (B) Dendrogram of gene clustering. Cluster dendrogram of genes based on topological overlap, with module colors assigned by dynamic tree cutting and merging of closely related modules. (C) Co‐expression module‐trait association heatmap. Module‐trait relationships displaying the correlation between gene modules and tumor status. The color scale represents the strength and direction of correlation, with corresponding *p*‐values in parentheses. (D) Gene significance vs. module membership in the red module. Scatter plot illustrating the relationship between module membership in the red module and gene significance for tumor status.

To precisely identify central genes associated with both cancer and ZNF384, we employed the WGCNA algorithm to construct a gene co‐expression network. We established a scale‐free co‐expression network with a soft threshold of 10, resulting in relatively good average connectivity (Figure 3A). Utilizing gene correlation, we constructed a hierarchical clustering dendrogram of genes. By setting the clustering height limit to 0.25, we merged highly correlated modules and identified eight similar gene modules (Figure 3B). We then examined the correlation between each module and clinical traits. Specifically, the red module, containing 331 genes, showed a positive correlation with the tumor (*r* = 0.51, *p* = 1e−23) (Figure 3C,D), leading us to designate the red module as clinically significant.

### 3.4 Gene Functional Analysis and Overlap Analysis

**FIGURE 4:**
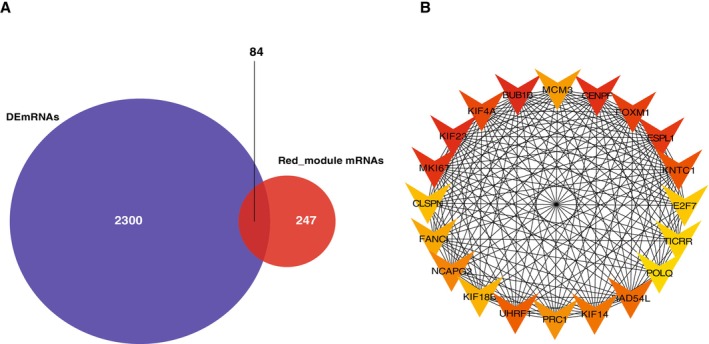
Network analysis and gene interaction visualization. (A) The overlap between red module genes and differentially expressed mRNAs (DEmRNAs). Venn diagram showing the overlap between red module genes (identified through WGCNA) and DEmRNAs. (B) Protein–protein interaction subnetwork of the top 20 hub genes, which were identified from the 84 overlapping candidates based on maximal clique centrality (MCC) scoring. The network was constructed using the STRING database (v11.5) and analyzed with the CytoHubba plugin in Cytoscape (v3.9.1).

**FIGURE 5:**
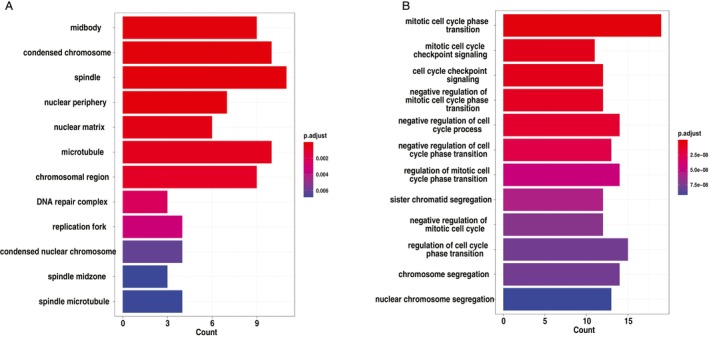
Functional enrichment analysis of overlap genes between DEGs and red module gene. (A) GO term enrichment analysis of cellular components. (B) GO term enrichment analysis of biological processes. The *x*‐axis represents the number of genes enriched in each term or process, while the color gradient represents the significance level (adjusted *p*‐value).

Subsequently, by overlapping the genes in the red module with DEGs, we identified a subset of 84 genes that are related to cancer and associated with ZNF384 (Figure 4A,B). To delve deeper into the possible mechanisms through which these 84 genes might impact COAD, we employed the R package “clusterProfiler” for gene function enrichment analysis, concentrated on their significant enrichment in biological processes (BP), molecular functions (MF), and cellular components (CC), as well as their involvement in key pathways such as DNA helicase activity, cell cycle, spindle, DNA repair, and replication fork (Figure 5). We also found that most of the 84 genes can be regulated by TP53, E2F1, SOX2, BRCA1, and MYC via TRRUST.

### 3.5 PPI Networks to Identify the Overlapped Genes

Utilizing the STRING database, we conducted a PPI network analysis on the 84 DEGs that overlapped with red module genes, then visualized it using Cytoscape V3.9.0. To further delineate the central players within this network, we employed the cytoHubba plugin in Cytoscape V3.9.0, extracting a contained top 20 genes subnetwork. This extraction process identified the following key genes as significant within the network: BUB1B, CENPF, CLSPN, E2F7, ESPL1, FANCI, FOXM1, KIF14, KIF18B, KIF23, KIF4A, KNTC1, MCM3, MKI67, NCAPG2, POLQ, PRC1, RAD54L, TICRR, and UHRF1. These genes are potentially crucial in understanding BP and pathways underlying the association between cancer and ZNF384.

Among them, FANCI, RAD54L, POLQ, and TICRR are DNA repair‐related genes that play crucial roles in HR, alternative end‐joining (alt‐EJ), and replication stress response, which are essential for maintaining genomic stability [36, 37, 38, 39]. BUB1B, CENPF, CLSPN, E2F7, FOXM1, and PRC1 are involved in cell cycle regulation, ensuring proper mitotic progression and checkpoint activation to prevent chromosomal instability [40, 41, 42, 43, 44, 45]. KIF14, KIF18B, KIF23, KIF4A, KNTC1, NCAPG2, and MCM3 regulate chromosomal integrity, mitotic spindle formation, and DNA replication, processes that are frequently dysregulated in colorectal cancer [46, 47, 48]. Lastly, MKI67, UHRF1, and ESPL1 are associated with tumor proliferation and epigenetic regulation, with UHRF1 contributing to DNA methylation and DDR‐related transcriptional control [49, 50, 51, 52].

### 3.6 Machine Learning Identification of Key Tumor‐Related Genes

**FIGURE 6:**
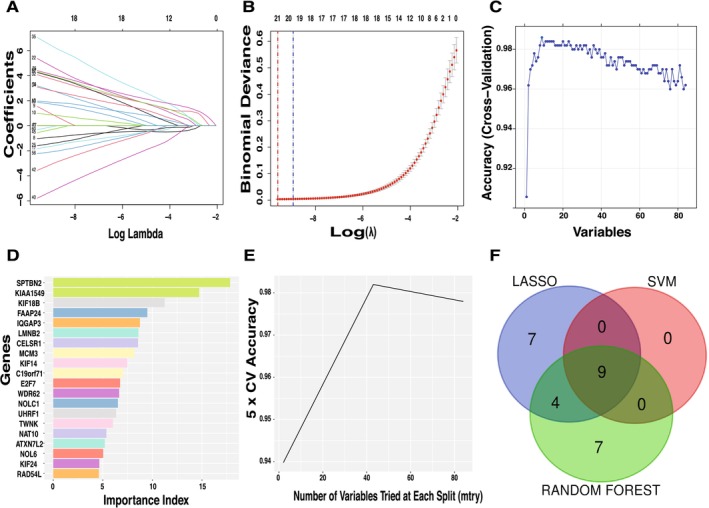
Feature selection and predictive modeling of key genes using LASSO, Random Forest, and SVM. (A) The Lasso regression path diagram. The trajectories of gene coefficients across different values of the regularization parameter (log *λ*). As *λ* increases, the coefficients of less informative genes are progressively shrunk to zero, allowing the selection of the most predictive features. The LASSO model identified 20 significant feature genes from the 84 overlapping candidate genes. (B) Cross‐validation curve for LASSO model selection. Binomial deviance is plotted against log(*λ*), with the optimal *λ* (blue dashed line) corresponding to the model with the lowest cross‐validation error. The one‐standard‐error rule *λ* (red dashed line) selects a more compact feature set. The numbers above indicate the remaining non‐zero coefficients at each *λ* value. (C) Cross‐validation accuracy vs. the number of selected variables. Model accuracy is plotted against the number of selected variables, showing performance stabilization as more features are incorporated. This indicates that a subset of genes contributes most to classification accuracy. (D) Random Forest feature importance ranking. The top 20 genes were ranked by their importance in classification accuracy. (E) Optimization of the Random Forest model. Fivefold cross‐validation (5 × CV) accuracy is plotted against the number of variables tried at each split (mtry). Model accuracy peaks at an optimal mtry, demonstrating the effect of variable selection on classification performance. (F) Consensus identification of key genes across machine learning models. A Venn diagram illustrating the overlap of genes selected by LASSO, Support Vector Machine (SVM), and Random Forest methods.

Utilizing LASSO, SVM, and Random Forest algorithms, we sought to pinpoint characteristic genes from a set of 84 overlapping candidate central genes. The LASSO method narrowed the field to 20 significant feature genes, while Random Forest analysis prioritized genes based on importance and selected the top 20. The SVM approach was more selective, identifying nine key genes (Figure 6A–E). Through a comprehensive intersection analysis of these methods, we consolidated our findings to nine most pertinent characteristic genes: KIAA1549, SPTBN2, IQGAP3, FAAP24, CELSR1, KIF14, LMNB2, KIF18B, and UHRF1 (Figure 6F).

### 3.7 ceRNA Network Analysis and Validation

**FIGURE 7:**
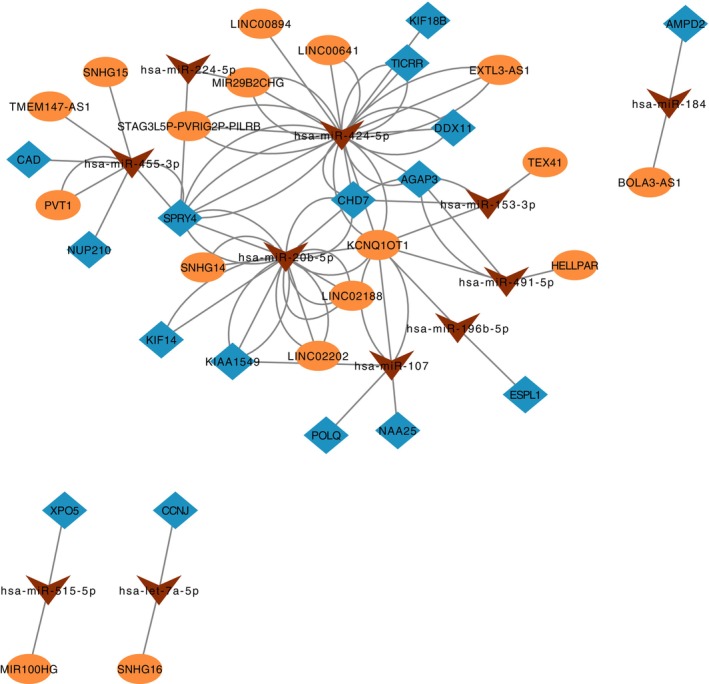
ceRNA network analysis of key tumor‐associated genes. Blue diamonds represent tumor‐associated mRNAs, orange ellipses indicate lncRNAs, and brown triangles denote miRNAs mediating lncRNA–mRNA interactions.

**FIGURE 8:**
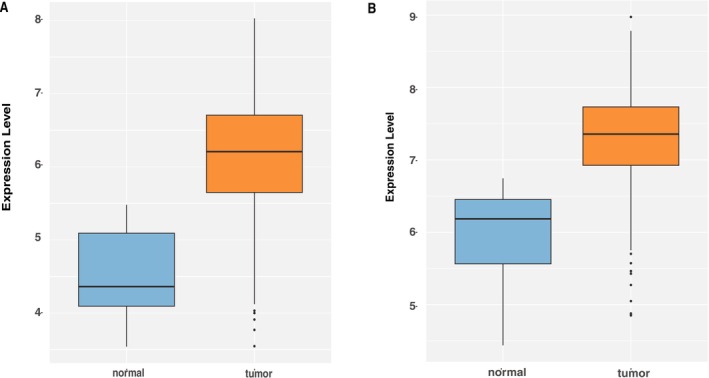
KIF14 and KIF18B expression level in GSE39582 data. (A) The boxplot of KIF14 showing the expression levels of KIF14 in normal and tumor samples. (B) The boxplot of KIF18B showing the expression levels of KIF18B in normal and tumor samples.

Based on the overlap of 84 above‐identified candidate core genes, we constructed a ceRNA network. An in‐depth analysis of this network revealed that KIF14 and KIF18B, genes pinpointed by previous LASSO, SVM, random forest, and PPI subnetwork analyses, also hold significant positions within the ceRNA network. They are regulated by two notable miRNAs, miR‐424‐5p and miR‐20b‐5p, which lie at the heart of the ceRNA network, exerting regulatory influence over mRNAs and lncRNAs intricately linked to the cell cycle (Figure 7). Notably, we discovered that several mRNAs and lncRNAs involved in cell cycle regulation directly connect to miR‐424‐5p and miR‐20b‐5p. This observation implies that these miRNAs could be pivotal in controlling the cell cycle through their impact on the expression of these mRNAs and lncRNAs. In addition, in validation analysis using the GSE39582 dataset, we identified a series of gene expression changes that were significantly associated with disease status. In particular, the regulatory relationship between the gene KIF14 and the gene KIF18B showed significant expression differences in disease samples compared with the control group (Figure 8).

## 4 Discussion

**FIGURE 9:**
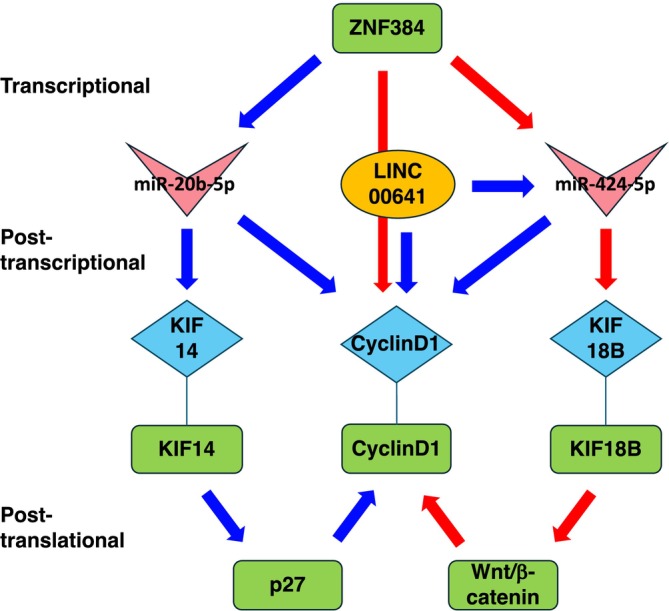
Suggested model for the regulatory network related to ZNF384. Protein, mRNA, miRNA, and lncRNA are shown with square, diamond, arrowhead, and ellipse, respectively. Red arrows and blue arrow indicate positive and negative regulation, respectively.

This study aimed to clarify the regulatory mechanisms involving ZNF384 in COAD. Toward this aim, we first identified a set of genes which are differentially expressed between tumor and normal tissues and are also related to ZNF384 using the TCGA dataset and then systematically integrated mRNAs, miRNAs, and lncRNAs into a unified ceRNA regulatory network. As a result, we identified miR‐20b‐5p, miR‐424‐5p, KIF14, and KIF18B as key components of this network, potentially driving COAD progression via coordinated modulation of cell cycle and mitotic pathways. miR‐20b‐5p and miR‐424‐5p were implicated in cancer either as tumor promoting factors or as tumor suppressing factors in various types of cancers [53, 54, 55, 56]. miR‐20b‐5p was shown to function as a tumor suppressor in colon cancer by targeting Cyclin D1 [57]. miR‐424‐5p was shown to promote proliferation and metastasis of colorectal cancer cells by targeting SCN4B [58] and also to inhibit cell proliferation by targeting E2F7, Cyclin E1, Cyclin A2, and Cyclin D1 [59, 60, 61, 62]. In addition, miR‐424‐5p is sponged by LINC00641, which also targets Cyclin D1 as well as c‐Myc and MMP2 [63]. At the post‐translational layer, KIF14 is shown to promote ubiquitination‐mediated degradation of p27, which is an inhibitor for cyclin‐dependent protein kinases [63, 64]. KIF18B is shown to activate the Wnt/β‐catenin pathway in cervical cancer, reducing Cyclin D1 expression [65]. Taken together, the present analysis highlights the intricate network of interactions among mRNA, lncRNA, and miRNA in COAD development and progression as illustrated in Figure 9. ZNF384 was shown to act as a direct transcriptional regulator of Cyclin D1, binding to its promoter region, in liver cancer [6]. Although ZNF384 itself is only modestly upregulated in COAD, these multiple pathways spanning multiple layers could reinforce the regulatory significance of ZNF384. In addition, our findings indicate that the upregulated mRNAs are primarily involved in DNA repair, replication fork stability, and cell cycle regulation, while the downregulated mRNAs may reflect suppressed tumor‐suppressive pathways. Furthermore, the upregulation of lncRNAs suggests their potential roles in ceRNA interactions, particularly in regulating key tumor‐associated genes. These expression changes reinforce the regulatory significance of ZNF384 in COAD progression and highlight the intricate network of interactions among mRNA, lncRNA, and miRNA in tumor development.

The factors found here could serve as biomarkers of COAD diagnoses and prognoses. From a therapeutic perspective, targeting miR‐20b‐5p and miR‐424‐5p may provide new intervention strategies. For example, miR‐20b‐5p upregulation has been linked to improved treatment responses [53], while miR‐424‐5p may influence chemotherapy sensitivity [53]. It is also noteworthy that both miR‐20b‐5p and miR‐424‐5p are implicated in diabetes. Metformin, a medicine to treat diabetes, is shown to upregulate miR‐20b‐5p [66, 67]. Conversely, saturated fatty acids, which potentiate insulin resistance, are shown to induce miR‐424‐5p [68]. Given their involvement in pathological processes, repurposing drugs and administering vitamins, such as vitamin E, as prophylactic agents with tumor‐modulatory effects may positively influence the examined genetic and epigenetic factors [69]. Furthermore, inhibiting KIF14 and KIF18B has been explored in other cancers, and future functional studies and drug sensitivity analyses will be essential to evaluate their potential as therapeutic targets.

A strength of our approach lies in its depth of network analysis, which has revealed ZNF384 as a previously unrecognized master regulator in colon cancer progression. Unlike previous studies that focused on isolated molecular interactions, our comprehensive network‐based methodology has illuminated a complex web of regulatory relationships, providing the first systematic characterization of ZNF384's role in cancer regulatory networks. Notably, our study is the first to establish the intricate interplay between ZNF384 and key microRNAs in the context of colon cancer, offering novel insights into tumor regulation mechanisms.

While our computational analyses provide these robust findings, we acknowledge limitations in our approach. Especially, experimental studies are warranted to validate interactions predicted in this study. Specifically, the modulation of miR‐20b‐5p and miR‐424‐5p by ZNF384 and the sponging role of LINC00641 need to be confirmed through functional assays, such as luciferase reporter assays and chromatin immunoprecipitation (ChIP) assays combined with knockdown and rescue of genes. Nevertheless, our methodology demonstrates how sophisticated data integration and analysis can reveal meaningful insights into complex cancer regulatory networks.

Looking forward, this work opens several promising avenues for future research in colon cancer biology. The novel regulatory relationships that we have identified, particularly those involving ZNF384 and its interaction with key microRNAs, provide a strong foundation for targeted experimental studies. Our findings suggest that ZNF384 plays a more central role in colon cancer progression than previously recognized, potentially serving as a crucial hub in the regulatory network that governs tumor development. The molecular interactions identified here offer new perspectives on potential therapeutic targets and underscore the importance of considering complex regulatory networks in cancer treatment strategies.

These findings significantly advance understanding of colon cancer regulatory networks while establishing a framework for future investigations. Our methodology demonstrates that sophisticated bioinformatics approaches, when carefully applied to existing datasets, can generate novel insights into cancer biology. The success of our approach in revealing complex regulatory relationships, particularly those centered on ZNF384, suggests that similar strategies could be effectively applied to study other aspects of cancer biology, potentially accelerating the pace of discovery in cancer research. Most importantly, our study highlights the value of examining cancer regulation from multiple angles, combining traditional gene expression analysis with network‐based approaches to uncover previously unrecognized molecular relationships that may be crucial for cancer development and progression.

## Author Contributions

**Bo Zhang:** conceptualization, data curation, formal analysis, investigation, writing – original draft. **Yoshihisa Matsumoto:** funding acquisition, supervision, writing – review and editing.

## Ethics Statement

The authors have nothing to report.

## Consent

The authors have nothing to report.

## Conflicts of Interest

The authors declare no conflicts of interest.

## Data Availability

The data that support the findings of this study are available in UCSC Xena at https://xena.ucsc.edu/public. These data were derived from the following resources available in the public domain: University of California Santa Cruz, https://xena.ucsc.edu.
